# Taguchi Optimization of Parameters for Feedstock Fabrication and FDM Manufacturing of Wear-Resistant UHMWPE-Based Composites

**DOI:** 10.3390/ma13122718

**Published:** 2020-06-15

**Authors:** Yury V. Dontsov, Sergey V. Panin, Dmitry G. Buslovich, Filippo Berto

**Affiliations:** 1 Laboratory of Mechanics of Polymer Composite Materials, Institute of Strength Physics and Materials Science SB RAS, 634055 Tomsk, Russia; doncov@mail2000.ru (Y.V.D.); buslovichdg@gmail.com (D.G.B.); 2 Department of Materials Science, Engineering School of Advanced Manufacturing Technologies, National Research Tomsk Polytechnic University, 634030 Tomsk, Russia; 3 Faculty of Engineering, Department of Mechanical and Industrial Engineering, Norwegian University of Science and Technology, 7491 Trondheim, Norway; filippo.berto@ntnu.no

**Keywords:** ultra-high molecular weight polyethylene, twin-screw extruder, compression sintering, compounding, mechanical properties, wear resistance, supermolecular structure, additive manufacturing, Taguchi method, 3D printing, Fused Deposition Modeling

## Abstract

It is believed that the structure and properties of parts fabricated by additive (i.e., non-stationary) manufacturing are slightly worse compared to hot pressing. To further proceed with improving the quality of Fused Deposition Modeling 3D-printed parts, the ‘UHMWPE + 17 wt.% HDPE-g-SMA + 12 wt.% PP’ composite feedstock fabrication parameters, by the twin-screw extruder compounding and 3D printing (the Fused Deposition Modeling (FDM) process), were optimized using the Taguchi method. The optimization was carried out over the results of mechanical tests. The obtained results were interpreted in terms of (1) the uniformity of mixing of the polymer components upon compounding and (2) the homogeneity of the structure formed by the 3D printing. The values of the main factors (the processing parameters) were determined using the Taguchi method. Their application made it possible to improve the physical, mechanical, and tribological properties of the samples manufactured by the FDM method at the level of neat UHMWPE as well as the UHMWPE-based composites fabricated by compression sintering. A comparative analysis of the structure, as well as the mechanical and tribological properties of the composite obtained by the FDM method, and the hot pressing from ‘optimized’ feedstock was performed. The ‘UHMWPE + 17 wt.% HDPE-g-SMA + 12 wt.% PP’ composites fabricated by the optimal compounding and 3D printing parameters can be implemented for the additive manufacturing of complex shape products (including medical implants, transport, mining, and processing industries; in particular, in the Far North).

## 1. Introduction

Two technologies are the most widely used 3D-printing methods with thermoplastic polymer materials: (1) Fused Deposition Modeling (FDM), and (2) Selective Laser Sintering (SLS). The FDM method is based on the layer-by-layer extrusion of molten polymer onto a heated part bed. The SLS method belongs to the class of the powder bed deposition technologies and is realized without vacuum by the local fusion of finely dispersed polymer powders using a laser beam. The main structural plastics used for 3D printing include thermoplastics with a melting point below 260 °C, such as acrylonitrile butadiene styrene, polylactic acid, polystyrene–butadiene–styrene, polyamide, polyvinyl alcohol, polycarbonate, etc. A lot of polyamide-based composites have been designed to manufacture products for tribological applications [1,2,3]. It is known that 3D printing with commercially available polyamides (PA-6, PA-66, and PA-12) is associated with a number of processing issues, in particular, thermal shrinkage, layer-by-layer post-printing delamination, etc. Nevertheless, designing thermoplastic matrix-based composites, including PA, is a promising way to produce parts for friction units.

Polyolefins (ultra-high molecular weight polyethylene (UHMWPE), polypropylene (PP), high-density polyethylene (HDPE), etc.) are less commonly applied to design feedstocks for 3D printing of the products [4,5,6] due to their low mechanical properties and melting point, as well as significant solidification shrinkage. Meanwhile, UHMWPE has several unique properties (a low friction coefficient, biocompatibility, high wear, and chemical resistance in aggressive environments), which determine its exceptional areas of applications. For this reason, UHMWPE is used in the friction units of machines and vehicles, as well as in medicine for the manufacturing orthopedic implants.

However, UHMWPE has an almost zero melt flow index (MFI < 0.06 g/10 min) because of its long polymer chains. This significantly limits the possibilities of its processing by methods typical for thermoplastic polymers, such as screw extrusion, injection molding, FDM, etc. For this reason, improving the processability of UHMWPE, as well as the UHMWPE-based composites, in terms of ensuring extrudability is relevant [7,8,9,10].

For instance, the HDPE-based composites have been manufactured by the FDM method, also known as fused filament fabrication (FFF) [11]. Their mechanical properties have been compared with the HDPE samples fabricated by injection molding. Well-known problems of shrinkage and interlayer adhesion, which are typical for HDPE processing by the FFF method, have been solved both by designing composites based on this polymer, and by adjusting the FFF parameters, such as temperature and a diameter of a nozzle, extrusion rate, temperature of a heated part bed, etc. The HDPE samples, obtained by the FFF method and injection molding, have possessed similar mechanical properties except for elongation at break. The authors have achieved high properties of the extruded polymer composite. This fact has been confirmed by both SEM images of the structure and tensile strength values.

The possibility of using the SLS method for manufacturing a special UHMWPE-based insert (implant) into the tibia has been studied in [12]. Its mechanical properties and dimensional accuracy of the applied procedure have been evaluated. The results have showed that it has been possible to improve ultimate tensile strength from 14.1 up to 24.1 MPa, and elongation at break from 5.4% up to 390% by controlling the shape using only post-printing heat treatment. In addition, it has been proposed to manufacture parts of the prostheses of the femoral and knee joints. However, the size of the manufactured products should be increased by 10.5% along the X and Y axes and by 6.5% along the Z axis.

In [13], an attempt has been made to overcome the problem of the low MFI of UHMWPE for the FDM method applying by its mixing with HDPE and polyethylene glycol (PEG). This has provided both satisfactory mechanical properties for biomedical applications and improved extrudability. It has been shown that loading with PEG has not been complied with conditions of direct mixing with UHWMPE, even if it has been a solid lubricant. In addition, loading UHMWPE with 60% HDPE has improved the mixture fluidity (according to MFI values). In this case, the composite has possessed acceptable thermal stability according to the results of thermogravimetric analysis (TGA).

The effect of the FDM parameters on the characteristics of the manufactured parts has been considered in [14]. The dependence of the mechanical properties from deposition algorithms, printing speed, filling degree (infill) and topography, extrusion temperature, and deposited layer heights has been studied. It has been shown that the dimensional accuracy of the product has been more affected by extrusion temperature, the deposition algorithms and the deposited layer heights compared with printing speed, as well as filling degree and topography. In addition, the mechanical properties have been highly dependent on the deposition algorithms, extrusion temperature, and the deposited layer heights. At the same time, with a high degree of filling (infill), its topography and print speed have had a little effect on the mechanical properties. Thus, the extrusion temperature and the deposited layer heights have been proposed to improve the mechanical properties.

In the review [15], it has been suggested to use a ‘UHMWPE + PP’ mixture as a material for the product manufacturing by the FDM method, as UHMWPE and PP possess proven effectiveness as biomaterials and are highly compatible with the human body. Polymer mixing has been viable in overcoming the UHMWPE extrudability issue. In this case, PP (as a material with a high MFI) is often applied to improve the rheological properties of polymers, although it is thermodynamically incompatible with UHMWPE.

In a previous paper by the authors [16], a three-component composite suitable for FDM printing has been designed. In addition, its structure, the mechanical properties, and the tribological properties have been studied. However, feedstock fabrication parameters have been predefined empirically. This paper describes the determination of the optimal parameters by the Taguchi method for both the twin-screw extruder compounding and 3D printing. The best parameter values have been found by assessing their physical and mechanical characteristics. In this case, ‘UHMWPE + 17 wt.% HDPE-g-SMA + 12 wt.% PP’ composite has been predefined according to preliminary studies. An analysis of the structure, the mechanical properties, and the tribological properties of the composites obtained by both the FDM method and compression sintering from the identical composition granules has been carried out to confirm the ‘optimality’.

Taguchi has developed a methodology for applying statistics to improve the quality of manufactured goods [17]. Since conducting full-scale (full factorial) experiments, as a rule, is expensive and takes a lot of time, it is necessary to reduce their number. From this point of view, the Taguchi method enables solving the problems of improving the quality of products (processing modes) with lower costs. For example, the authors of the paper [18] have found the optimal parameters of electron beam melting of a titanium alloy by the Taguchi method to improve the surface quality (reduce roughness) using subsequent ultrasonic treatment.

The Taguchi method has been used widely enough to optimize 3D-printing parameters [19,20,21,22,23,24,25,26]. In [26], the effect of five parameters of the FDM method has been investigated. They have included the deposited layer height, the deposition algorithms, the sample orientation, air gap, and raster width. Additionally, their optimal combination has been found by the Taguchi L27 orthogonal matrix. Experimental results have showed that the sizes of the printed products always exceed those specified in a control program. However, the length, width, and diameter of holes in the samples have been always less than given values. It has been necessary to preset optimal coefficient values for each characteristic, namely, the percentage change in length, width, thickness, and diameter. The gray-Taguchi method has been applied in [26] to minimize the variation of all four parameters at the same time. The optimal factor levels have been found: the layer thickness has been 0.254 mm, the sample orientation has been 0 degrees, the raster angle (the angle measured from the layer X horizontal axis to a raster) has been 0 degrees, the raster width (the width of the material bead used for the raster’s) has been 0.4564 mm; and the air gap (the distances (spaces) between two adjacent raster’s) has been 0.008 mm.

Thus, the aim of this work has been to determine the suitable (optimal) parameters of (1) the twin-screw compounding, and (2) the FDM manufacturing of the ‘UHMWPE + 17 wt.% HDPE-g-SMA + 12 wt.% PP’ composite to ensure the best mechanical and tribological properties of the products.

## 2. Materials and Methods

**Figure 1:**
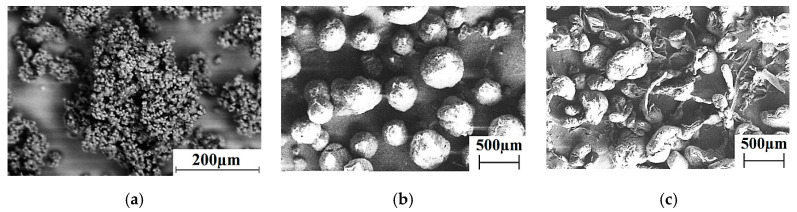
Micrographs of the ultra-high molecular weight polyethylene (UHMWPE) (**a**), polypropylene (PP) (**b**), and high-density polyethylene grafted with maleic anhydride (HDPE-g-SMA) (**c**) powders.

The ‘Ticona GUR-2122’ UHMWPE powder (Celanese Corporation, Irving, TX, USA) was used with a molecular weight of 4.5 million and an individual particle size of 5–10 μm in the form of slightly agglomerated particles 130 μm in size (Figure 1a). High-density polyethylene grafted with maleic anhydride (HDPE-g-SMA; milled granulate, particle sizes of about 500 μm shown in Figure 1c, ‘New Polymeric Technology’ LLC, Unecha, Russia) and the ‘PP21030’ polypropylene powder (MFI = 3.0 g/10 min, average particle size of 600 μm presented in Figure 1b, ‘Tomskneftechim’ LLC, Tomsk, Russia) were loaded as plasticizing additives.

Preliminary dry mixing of the UHMWPE and polymer modifier powders was carried out in a ‘MP/0.5×4’ planetary ball mill (‘Technocenter’ LLC, Rybinsk, Russia). Then, the polymer binder powder and the fillers were mixed by dispersing the suspension components in alcohol using a ‘PSB Gals 1335-05’ ultrasonic cleaner (‘PSB-Gals’ Ultrasonic equipment center, Moscow, Russia). The processing time was 3 min; the generator frequency was 22 kHz. After mixing, a suspension of the components was dried in an oven with forced ventilation for 3 h at a temperature of 120 °C.

In order to homogenously mix small UHMWPE particles (tens of microns in size) and large particles of the polymer fillers (hundreds of microns), they were compounded with a ‘Rondol’ twin-screw extruder (10 mm Twin Screw Extruder, Microlab, Rondol, France).

Variable parameters (factors) upon compounding were the following:

(1) The extruder screw rotation speed (υ_extr2scr_) was 40 rpm, 50 rpm, and 60 rpm. The rotation of both extruder screws was at the same speed in a clockwise direction. A heated area was along a barrel that included five zones: the first one was cooled with distilled water, and the last (die zone) was equipped with a pressure sensor and a die nozzle with a diameter of 2 mm. The polymer powder mixture was uniformly moved from a hopper to a feeder of the twin-screw extruder using an unheated single-screw one. This limited the amount of the supplied powder.
(2) The extrusion temperature (*T*_extr2scr_) was preset at three levels: 210 °C, 220 °C, and 230 °C. Its values varied simultaneously with temperature of the five heated zones near the die nozzle (Table 1).
(3) The amount of the extruded material (N_extr_) was preset as 1, 2, and 3 passes. After each pass, the extrudate was mechanically chopped using a ‘Rondol’ chopper (Rondol, France) to granules (pellets) with a size of 2 × 3–4 mm (where 2 mm was the diameter corresponding to that of the output extruder nozzle).

Then, bulk preforms of the polymer composites were fabricated in two ways:

(a) By the hot compression of the granules. The three-component mixtures were processed at a pressure of 10 MPa and a temperature of 200 °C using a laboratory setup based on a ‘MS-500’ hydraulic press (NPK TekhMash LLC, Moscow, Russia). The setup was equipped with an open-loop ring furnace with digital temperature control (ITM LLC, Tomsk, Russia). After holding under pressure, the preforms were cooled without unloading for 30 min. The cooling rate was 5 °C/min.
(b) By the FDM method. Rectangular samples with a height of 10 mm were manufactured from the granules of the same polymer mixtures using an ‘ArmPrint-2’ homemade printer (Tomsk Polytechnic University, Tomsk, Russia). The 3D printer was equipped with a single-screw micro-extruder with a nozzle diameter of 0.4 mm for printing with granules with a size (diameter) of 1–5 mm. The ‘ArmPrint-2’ setup moved the print head along the XYZ axes. The amount of the supplied material was determined by the micro-extruder screw rotation speed. The 3D printer was equipped with a heated part bed having a temperature variation range of 20–300 °C. The temperature range for the material heating in the micro-extruder was from 20 up to 420 °C. Control was provided using the ‘LINUX CNC’ operating system. Three-dimensional printing was based on a model designed in the ‘G-code’ format. Digital model files were created using the ‘Repetir-Host V2.1.3’ software (Hot-World GmbH & Co. KG Knickelsdorf 4247877, Willich, Germany) and the ‘Slic3r’ slicer (licensed under the GNU Affero General Public License, version 3). The software parameters were preset as the following: the layer height was 0 mm; the first layer height was 0.3 mm; the perimeters were 3; the top solid layer was 0 and the bottom one was 0; the infill was 100%; the rectilinear and speed were the same, but the first layer was 50%; and the extrusion width infill was 250%.

The variable parameters (factors) for the FDM methods were the following:

(1) The micro-screw rotation speed (*f*_3D_) was preset in terms of [rpm] at an equal material supply rate, which corresponded to the predefined coefficient in the 3D printer control software with values of 7.7, 8.0, and 8.3; or, respectively, 0.196, 0.202, and 0.211 revolutions per second that were equal to 11.76, 12.12, and 12.66 rpm.
(2) Part bed temperature (*T*_3Dbed_) was 100, 110, and 120 °C.
(3) Printing speed (*v*_3D_) was 10, 20, and 30 mm/s.
(4) Micro-extruder temperature (*T*_3Dextr_) was 170, 180, and 190 °C.

Specimens for tensile and wear tests were made from the samples manufactured by the FDM method (70 mm × 70 mm × 10 mm) using a milling machine.

Tensile properties of the ‘dog-bone’ shaped UHMWPE-based specimens were measured under tension using an ‘Instron 5582’ electromechanical testing machine (Instron, Norwood, MA, USA). There were at least four of each type of sample.

The ‘Pin-on-disk’ dry sliding friction tests were performed to determine the friction coefficients using a ‘CSEM CH-2000’ tribometer (CSEM, Neuchâtel, Switzerland). The load was 5 N; the contact pressure P_max_ was 31.8 MPa; and the sliding speed was 0.3 m/s. A ball-shaped counterpart 6 mm in diameter was made of the GCr15 bearing steel (the distance was 1 km; the track radius was 10 mm; and the rotation speed was 286 rpm).

The wear resistance was evaluated according to the “block-on-ring” scheme using a “2070 SMT-1” friction testing machine (Tochpribor Production Association, Ivanovo, Russia). The load on the samples was 60 and 140 N; (estimated) contact pressure P*_max_* was 9.7 and 32.4 MPa; sliding speed was 0.3 and 0.5 m/s. A counterpart was made of the outer ring of a commercial bearing. It had a disk shape with a diameter of 35 mm and a width of 11 mm. The counterpart surface roughness was 0.20–0.25 µm. The wear rate was determined by measuring the width and depth of the wear track according to stylus profilometry, followed by multiplication by its length. The wear rate values were calculated considering the data on the applied load and the sliding distance:

(1) $$\mathrm{Wear}\;\mathrm{rate}\;=\frac{\mathrm{volume}\;\mathrm{loss}\;\left({\mathrm{mm}}^{3}\right)}{\mathrm{load}\left(N\right)\cdot \mathrm{sliding}\;\mathrm{distance}\;\left(m\right)}.$$

The wear track profiles were determined using the data on at least 10 tracks. Then, the wear rate values were estimated based on the experimental test data over at least four samples of each type. Mathematical statistics methods were used for the experimental results processing.

The surface topography of the wear tracks was studied using a “Neophot-2” optical microscope (Carl Zeiss, Oberkochen, Germany) equipped with a “Canon EOS 550D” digital camera (Canon Inc., Tokyo, Japan) and an “Alpha-Step IQ” contact profiler (KLA-Tencor, Milpitas, CA, USA).

The cleaved surfaces of the notched specimens mechanically fractured after exposure in liquid nitrogen were used for supermolecular structure studies. A “LEO EVO 50” scanning electron microscope (Carl Zeiss, Oberkochen, Germany) was employed (accelerating voltage was 20 kV).

IR spectra were recorded using ‘NICOLET 5700’ (Thermo Fisher Scientific, Waltham, MA, USA) and ‘FT-801’ (SIMEX, Novosibirsk, Russia) Fourier-transform IR spectrometers in the range of 600–4000 cm^−1^ diffuse reflectance with a diamond (Single Reflection Diamond ATR).

The mechanical tests were carried out to determine the key properties according to [27]. The tribological tests, which were focused primarily on assessing the wear resistance, were performed according to [28,29]. The sliding speed of 0.3 m/s was set the same for both applied test schemes (‘pin-on-disk’ and ‘block-on-ring’). It should be noted that the installation, available to the authors, for the tribological testing in accordance with the ‘block-on-ring’ scheme did not enable evaluating the friction coefficient. To solve this problem, the ‘CSEM CH-2000’ tribometer was applied at the load of 5 N. As a result, the volumetric wear of the UHMWPE-based composites was evaluated in the steady wear mode. According to the published data, the sliding speed of about 0.3 m/s was used quite often [30]. Therefore, the results obtained by the authors were comparable. In addition, the wear resistance and the friction coefficient of neat UHMWPE were investigated [31] according to the ‘block-on-ring’ scheme with load variations from 40 to 160 N. It was shown that the friction coefficient decreased from 0.051 to 0.040, but the volumetric wear enhanced with load increasing.

## 3. Experimental Results

### 3.1. Variation of the Twin-Screw Compounding Parameters

Since the compounding in the twin-screw extruder enabled distributing the components most uniformly, the following factors were used as the variable ones: (1) extrusion temperature *T*_extr2scr_, (2) the extruder screw rotation speed *v*_extr2scr_ (determining the extrusion rate, i.e., the amount of the supplied material), and (3) the amount of the extruded material *N*_extr_ (the amount of the mixed material). In this case, the mixing temperature was determined by the mixture viscosity. Its rise intensified the mobility of macromolecule segments, enhancing the mutual penetration of the polymers. An increase in the extruder screw rotation speed had to intensify the mixing of the components as well. In addition, based on the assumption that the extremely low melt flow rate (MFI) of UHMWPE did not enable it to be effectively mixed with the plasticizing components, an increase in uniformity had to be achieved by rising the number of passes of the supplied mixture through the twin-screw extruder.

Then, the number of levels of all analyzed factors (the processing parameters) and the range of their possible changes were estimated.

I. The temperature range was predefined from 210 up to 230 °C because the polymer mixture was very viscous at lower temperatures that made it difficult to process by extrusion (reducing, first, extrusion rate). On the other hand, higher temperatures could result in a wider molar mass distribution of the polymers due to the UHMWPE and HDPE-g-SMA thermal degradation. This could cause the deterioration of their physical and mechanical (including tribological) characteristics.
II. The variation range of the extruder screw rotation speed was determined based on the extruder performance, as well as the visually controlled uniformity of the polymer mixture extrudate flow.
III. Finally, the number of passes (mixing) of more than three was time-consuming and not economically feasible.

The data were summarized according to the Taguchi method in Table 2 combining the factors and their levels. Its feature was that all levels and factors met three times. Thus, nine granulate types of the same composition were prepared under different conditions. Then, plates 70 mm × 70 mm × 10 mm in size were fabricated by hot pressing, and the ‘dog-bone’ specimens were cut using a milling machine for the tensile tests, the results of which are presented in Table 3. Statistical processing of the results was carried out using four samples. The confidence interval was evaluated, and one value was discarded then. Thus, the mechanical properties of three samples were used by the Taguchi method analysis.

The Taguchi formula (bigger is better) was used to identify the optimal levels of the factors (the processing parameters) of the twin-screw compounding:

(2) $$\mathrm{MSD}\;=\left(\sum \frac{1}{y_{n}^{2}}\right)/n$$

(3) S/N =−log10(MSD)

where

*n* was the number of repetitions.
*y* was the result of the experiments.
MSD was the mean square deviation from the target value of the quality characteristic.
S/N was the variance index called the signal-to-noise (S/N) ratio.

Then, the S/N parameter was determined by summing the obtained values at one factor level. A more significant difference between the S/N values at one level compared to another indicated a greater effect of the analyzed factor. This difference was classified as the Delta parameter (or *L*_max_ – *L*_min_, i.e., the difference in the S/N values between the maximum and minimum factor levels). This value reflected the contribution of the parameter to the change in the values of the physical and mechanical properties during the transition from one level to another. As a result, a factor that varied over a larger range of values resulting in a more noticeable variation in the physical and mechanical characteristics was determined.

For example, according to the obtained data, the difference between the extruder screw rotation speed *v*_extr2scr_ levels affected the Young’s modulus to a greater extent than the extrusion temperature *T*_extr2scr_ and the amount of the extruded material *N*_extr_. Thus, the Young’s modulus value was changed more significantly when the ‘the extruder screw rotation speed *v*_extr2scr_’ factor shifted from one level to another than when the ‘extrusion temperature *T*_extr2scr_’ factor was the same.

**Figure 2:**
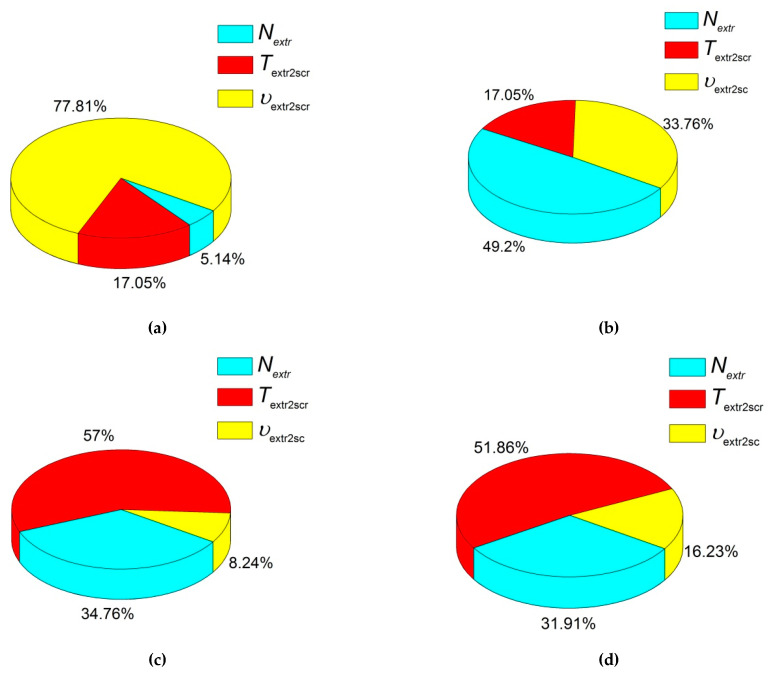
The pie charts of the effect of the factors on Young’s modulus (**a**), yield strength (**b**), tensile strength (**c**), and elongation at break (**d**) of the composites compounded by the twin-screw extrusion.

These results are shown in Figure 2a. They characterized the effect degree of the processing factors considering the levels of their change. The maximum effect on Young’s modulus was exerted by ‘the extruder screw rotation speed *v*_extr2scr_’, while the total effect of ‘the amount of the extruded material *N*_extr_’ and ‘extrusion temperature *T*_extr2scr_’ factors did not exceed 25%. Accordingly, the extruder screw rotation speed had to be the maximum to achieve the highest Young’s modulus value. It is highly likely that this was associated with an increase in pressure and, respectively, an intensification of intermolecular interaction and better dispersion of the components. Figure 2b–d present pie charts for other physical and mechanical characteristics and the effect of the factors on them.

**Figure 3:**
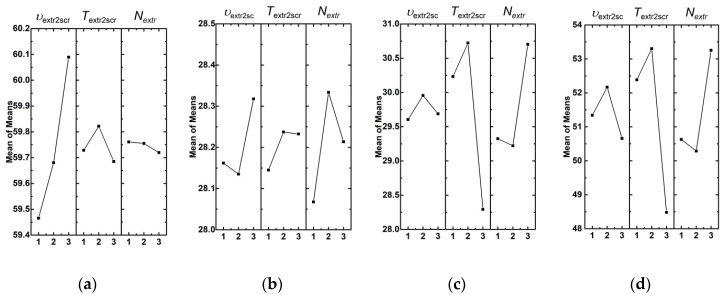
The effect of the factors on Young’s modulus (**a**), yield strength (**b**), tensile strength (**c**), and elongation at break (**d**) of the composites compounded by the twin-screw extrusion.

A line graph in Figure 3a additionally illustrates the effect of the factor levels (the processing parameters) on Young’s modulus. An increase in the extruder screw rotation speed from 40 and 60 rpm resulted in enhance Young’s modulus by approximately 1% (from 987 up to 1000 MPa). Other factors changed without a pronounced trend and in a much lower range of the values. Thus, from the point of view of Young’s modulus, the most significant factor was the extruder screw rotation speed, the value of which was supposed to be at its maximum.

The next analyzed parameter was yield strength. The corresponding experimental data for varying the factors and their levels are presented in Figure 2b. It follows from the pie chart analyses that the greatest effect on yield strength had the amount of the extruded material. Therefore, precisely the multiple re-mixing of the polymer mixture in the extruder resulted in the improvement of the composite yield strength. At the same time, the extruder screw rotation speed was a less significant parameter. The extrusion temperature had a minimal effect. On the line graph in Figure 3b, the nature of these effects could be seen. It follows from these data that twofold mixing caused the highest yield strength values, while threefold processing slightly reduced it. A possible reason could be the multiple thermal cycles of the polymer mixture, as well as the local polymer damages due to the grinding of the extrudate. Nevertheless, the high extruder screw rotation speed resulted in the yield strength improvement. Therefore, insufficient efforts at the low extruder screw rotation speed did not enable forming the required structure homogeneity of the polymer mixture.

The next analyzed parameter was tensile strength (Figure 2c and Figure 3c). It was shown that the extrusion temperature exerted the greatest effect on tensile strength. Its optimal value was 220 °C according to the data obtained by the Taguchi method. It is highly likely that this was since the mixing of the polymer components was insufficient at lower temperatures, while it was accompanied by the degradation and/or oxidation processes at higher ones. A slightly less significant factor was the amount of the extruded material. According to the obtained data, tensile strength was improved when the number of passes was more than one. This was consistent with the idea of a better polymer mixing and the formation of a more homogeneous structure.

The last but not the least analyzed parameter was elongation at break (Figure 2d and Figure 3d). It was shown that the extrusion temperature had the greatest effect on it. According to the authors, this was since the extrusion temperature of 220 °C was sufficient for uniform mixing and the formation of minimum internal defects (flaws). The second most important factor, that had the effect on elongation at break, was the amount of the extruded material, since the distribution of the components was more uniform. This enabled forming a homogeneous UHMWPE matrix structure. The least effect on elongation at break (about 20%) was exerted by the extruder screw rotation speed. Based on the obtained data, its optimal value was 50 rpm.

Summing up the intermediate result, it can be concluded that the optimal twin-screw compounding temperature was the maximum studied value of 220 °C. In this case, the strength characteristics such as Young’s modulus and tensile strength were the highest. On the other hand, the optimal extruder screw rotation speed was between 50 and 60 rpm. At lower values, the physical and mechanical properties of the composites had been deteriorated.

The amount of the extruded material had a controversial effect. There were significant changes in yield strength, tensile strength, and elongation at break with repeated mixing, while the Young’s modulus changed only slightly. From the standpoint of estimating the amount of the extruded material, the assessment of the most suited values was the result of a compromise between the time spent on the mixing and the mechanical characteristics of the composites. It can be concluded by analyzing the graphs in Figure 3 that the single powder mixture processing in the twin-screw extruder did not enable obtaining high-quality mixing.

The obtained results are summarized in Table 4, where the numbers indicate significance, and below are the optimal values of the factors.

### 3.2. Variation of the FDM Process Parameters

The granulate (pellets) fabricated by compounding in the twin-screw extruder at the extruder screw rotation speed *v*_extr2scr_ of 50 rpm, at a temperature *T*_extr2scr_ of 220 °C, and the amount of the extruded material of one was used to manufacture the samples (70 mm × 70 mm × 10 mm) in the amount of nine pieces varying the FDM process parameters for each sample. Unfortunately, these experiments were carried out simultaneously with studies on the optimization of compounding parameters (described in the previous section). Therefore, the applied compounding parameters were not optimal.

The factors that had the greatest effect on the physical and mechanical characteristics of the composites were chosen (both according to the authors and the published data [32,33]).

I. The micro-extruder screw rotation speed (*f*_3D_) directly affected the amount of the extruded material. This factor was determined as the amount of the material “squeezed out” from the 3D-printer nozzle per meter of a deposited bead. This parameter was preset in the ‘Slic3r’ software (‘Extrusion multiplier’ section) as a dimensionless coefficient. It had been previously established that the value of this parameter (factor) had to exceed 7.7 at 100% ‘Infill’ to manufacture the samples from the studied polymer mixture and maintain the required shape. At lower values, internal cavities and defects had been formed, but excess material had been accumulated on the manufactured samples at a magnitude of this factor above 8.5. This had been accompanied by the formation of sagging, an increase in the roughness of the wall surfaces, as well as the sample shape distortion from the predefined parallelepiped. In these investigations, the micro-extruder screw rotation speed *f*_3D_ was 11.76, 12.12, and 12.66 rpm (in terms of the control software, the coefficients were 7.7, 8.0, and 8.3).
II. The following significant processing factors were part bed temperature (*T*_3Dbed_) and micro-extruder temperature (*T*_3Dextr_). Both factors affected the formation of the composite structure and the cohesion between the deposited layers. The values of micro-extruder temperature were preset based on the melting points of the polymer mixture components. Since polypropylene had the highest melting temperature (from 130 up to 170 °C), the micro-extruder temperature levels were also preset as exceeding it by tens of degrees (*T*_3Dextr_ = 170; 180 and 190 °C).
III. The upper part bed temperature was limited to 120 °C. At higher temperatures, the material surfaces were oxidized and turned yellow. However, the samples were distorted upon printing at a part bed temperature of about 80–90 °C. The reason was the alteration of the thermal expansion coefficients of the layers heated to different temperatures. The part bed temperature *T*_3Dbed_ levels were 100, 110, and 120 °C.
IV. The fourth processing factor was printing speed (*v*_3D_), i.e., the movement of the micro-extruder head relative to the part bed. In general, the value of this parameter determined the additive manufacturing performance. Therefore, the finding out the most suited value was a compromise between the process efficiency and the uniformity of the formed macro- and microstructure. The main limiting parameter of this processing factor was the polymer mixture viscosity, which did not enable supplying the required amount of the molten polymer on a build platform. For this reason, the samples manufactured by the non-optimized FDM method were characterized by high porosity at printing speeds *v*_3D_ above 30 mm/s.

Based on the mentioned criteria, the factor levels were chosen (changes in the processing parameters, Table 5).

Then, by analogy with the previous section, an analysis was carried out of the measured values of the physical and mechanical properties of the samples manufactured by the conventional FDM method, the total number of which for the tensile tests was 36. Additionally, their density and shore (D) hardness was measured. These characteristics, as well as the results of the mechanical tests of the samples manufactured by the non-optimized FDM method, are presented in Table 6. Statistical processing of the results was carried out using four samples with the confidence interval calculation.

#### 3.2.1. Young’s Modulus

**Figure 4:**
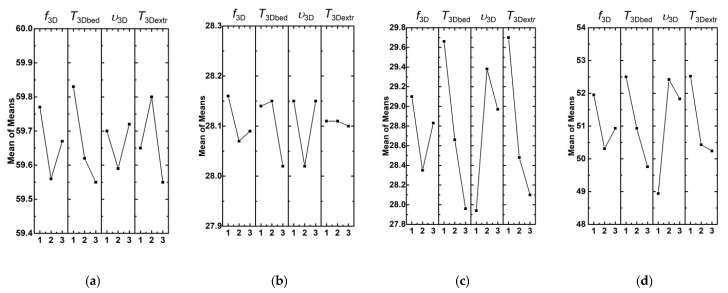
The effect of the factors on Young’s modulus (**a**), yield strength (**b**), tensile strength (**c**), and elongation at break (**d**) of the composites manufactured by the non-optimized FDM method.

**Figure 5:**
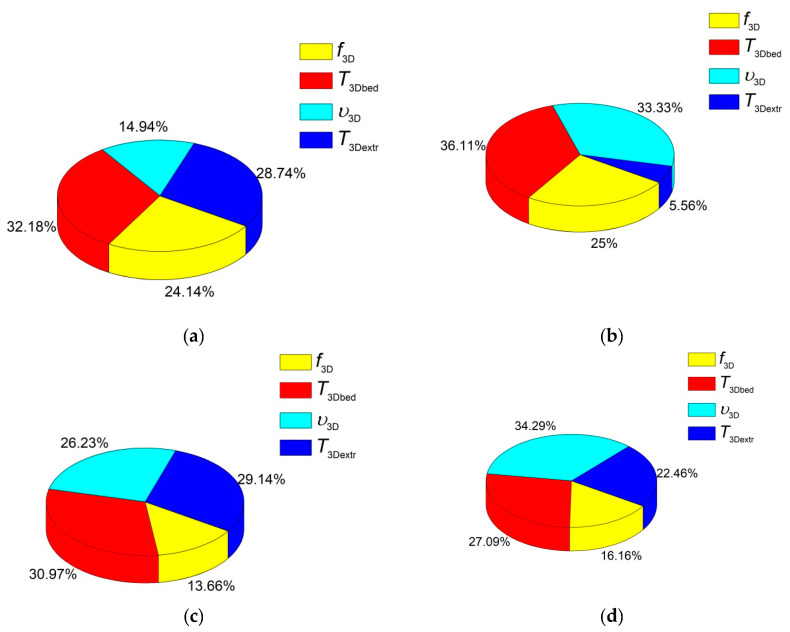
Pie charts of the effect of the factors on Young’s modulus (**a**), yield strength (**b**), tensile strength (**c**), and elongation at break (**d**) of the composites manufactured by the non-optimized FDM method.

Figure 4 shows dependences of Young’s modulus, tensile strength, yield strength, and elongation at break in terms of the average ‘signal-to-noise’ (S/N) sum (mean of the means) with varying levels of the processing factors. The greatest effect on Young’s modulus had the bed temperature *T*_3Dbed_ (32.2%). Less significant factors were the micro-extruder temperature *T*_3Dextr_ and the micro-extruder screw rotation speed *f*_3D_, (28.7% and 24.1%, respectively; Figure 5a). The least significant in the studied range of variation was printing speed *v*_3D_ (14.9%; Figure 5a). It should be noted that the effects of all factors on the Young’s modulus were comparable in general.

It can be concluded by analyzing the results (the factor levels) that that the minimum values had to possess part bed temperature *T*_3Dbed_ (approximately 100 °C), micro-extruder temperature *T*_3Dextr_ (180 °C), and the micro-extruder screw rotation speed *f*_3D_ (11.76 rpm). Printing speed did not show a clear effect.

#### 3.2.2. Yield Strength

The yield strength (Figure 4b and Figure 5b) was almost equally affected by two processing factors: part bed temperature *T*_3Dbed_ (the effect degree was 36%) and printing speed *v*_3D_ (the effect degree was 33%). Moreover, the ‘average’ value of part bed temperature (not higher than 110 °C) did not cause the distortion and oxidation of the samples. This made it possible to obtain high physical and mechanical properties of the composites. It is suggested that the samples upon printing had a temperature slightly lower than the heated part bed. It should be noted that the samples were constantly in direct contact with atmospheric oxygen upon 3D printing. Therefore, the oxidation processes were possible at high temperatures. This conclusion was also confirmed by the results of IR spectroscopy. The micro-extruder screw rotation speed *f*_3D_ had a lesser effect (25%). The micro-extruder temperature had a very slight effect *T*_3Dextr_ (<6%).

#### 3.2.3. Tensile Strength

Figure 5c shows the dependences of the tensile strength values from four processing factors and their levels. Part bed temperature *T*_3Dbed_, micro-extruder temperature *T*_3Dextr_, and printing speed *v*_3D_ improved tensile strength (the given order of the factors corresponded to their effect degree). The micro-extruder screw rotation speed *f*_3D_ affected to a lesser extent. The highest tensile strengths were at the parts bed temperature *T*_3Dbed_ of 100 °C and the micro-extruder temperature *T*_3Dextr_ of 170 °C (Figure 4c). Their effects were 31.0% and 29.1%, respectively. The printing speed *v*_3D_ had to be 20 mm/s (26.2%; Figure 5c). However, the weak significance of the micro-extruder screw rotation speed *f*_3D_ factor (13.7%) testified to its lesser contribution for providing high tensile strength. According to the authors, this factor had to possess a higher effect degree in the case of a wider range of its values.

#### 3.2.4. Elongation at Break

According to the data given in Figure 4d and Figure 5d, elongation at break was most affected by printing speed *v*_3D_ (34.3%). The maximum elongation at break was achieved at the printing speed of 20 mm/s (Figure 4d; level 2). Then, as the effect degree decreased, they were ranked: part bed temperature *T*_3Dbed_, micro-extruder temperature *T*_3Dextr_, and the micro-extruder screw rotation speed *f*_3D_ (Figure 5d; 27.1%, 22.5%, and 16.2%, respectively). The highest value of elongation at break was achieved with the following processing factors: the part bed temperature *T*_3Dbed_ of 100 °C and the micro-extruder temperature *T*_3Dextr_ of 170 °C. These data are summarized in Table 7.

#### 3.2.5. Summary Data

To determine the processing factors, the change in the levels of which had caused a more significant improvement of the physical and mechanical properties, a summary Table 7 was compiled with a point assessment system. The factor that had the maximum effect degree was marked by the minimum number. As a result, the factor that contributed to the greatest effect was determined from the analyzed values of the physical and mechanical properties. This factor was part bed temperature *T*_3Dbed_. Lower, but close in magnitude, were the printing speed *v*_3D_ and micro-extruder temperature *T*_3Dextr_. A possible reason for this was the low difference in the factor values at each level. It is highly likely that these factors could possess a much more significant role if their micro-extruder temperature levels had been changed with a double step (for example, 160, 180, 200 °C). For this reason, the micro-extruder screw rotation speed *f*_3D_, which manifested as a factor that had had a slight effect, was only in fourth rank in terms of the effect degree precisely due to the similarity of the values of neighboring levels. However, the samples were porous at lower the micro-extruder screw rotation speed values, and their physical and mechanical properties were low.

To determine the optimal FDM process parameters, the highest S/N values were assessed relative to their levels for each factor. In Table 7, these were presented as the optimal values. As a result of the systematization, it was shown that the highest physical and mechanical properties of the samples manufactured by the conventional FDM method had been achieved at the part bed temperature *T*_3Dbed_ of 100 °C, the printing speed *v*_3D_ of 20 mm/s, and the micro-extruder temperature *T*_3Dextr_ of 170 °C. The micro-extruder screw rotation speed *f*_3D_ (the amount of the supplied material), as a factor that had a minimal effect degree had to be taken equal to the average value of 12.12 rpm.

## 4. Verification of the Determined Optimal Values of the Processing Parameters

Two samples in the form of a parallelepiped (50 mm × 50 mm × 10 mm fabricated by compression sintering of the granulate (pellets) and 70 mm × 70 mm × 10 mm manufactured by the FDM method) were obtained using the determined optimal parameters of the twin-screw compounding and the FDM method. Specimens for studies of the structure, the mechanical properties, and the tribological properties were cut from them by a milling machine. The results are given below.

### 4.1. Structure Characterization Results

**Figure 6:**
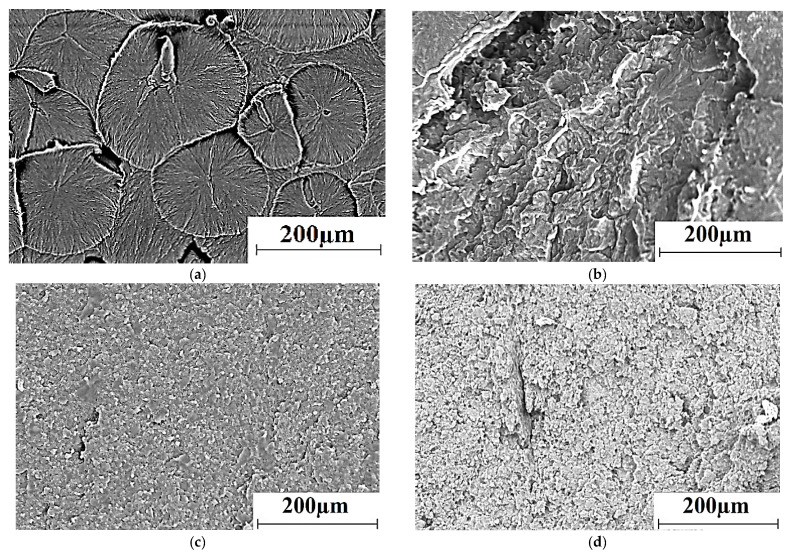
The supermolecular structure of neat UHMWPE (**a**) and the ‘UHMWPE+17 wt.% HDPE-g-SMA +12 wt.% PP’ composite; non-optimized powder hot pressing (**b**), non-optimized granule hot pressing (**c**), non-optimized FDM method (**d**); ultra-high molecular weight polyethylene (UHMWPE), polypropylene (PP), high-density polyethylene (HDPE).

Figure 6 shows SEM micrographs of the supermolecular structure of neat UHMWPE and the composites obtained by compression sintering of the powders and the granules, as well as by the FDM method. The structure of the sample fabricated by the conventional hot pressing of the neat UHMWPE powder had a spherulite pattern with a characteristic size of structural elements of 100–200 μm (Figure 6).

The structure was extremely heterogeneous in the case of hot pressing of the powder mixture (Figure 6b). First, this was due to the large size of the initial PP powder particles (hundreds of microns), which had a pronounced interface with the UHMWPE matrix obtained by powder compression sintering. In addition, PP particles were presented as separate inclusions of a rounded shape due to the difference in melting/solidification temperatures (not less than 160 °C). A significant number of discontinuities at phase interfaces, formed during compression sintering, had a decisive role in reducing the physical and mechanical properties of the samples fabricated from the powder (as shown below).

Figure 6c,d show SEM micrographs of the microstructure of the composites manufactured by compression sintering and the FDM method from the granulate after the twin-screw extrusion. The high degree of the structure uniformity of the polymer composite under conditions of the effective polypropylene (as well as HDPE-g-SMA) dispersion in the UHMWPE matrix should be noted. This effect had been realized due to compounding the mixture in the twin-screw extruder, as well as the difference in polyethylene and PP melting points. However, the spherulite structure had not been formed in these cases. In addition, the structure of the composite fabricated by compression sintering was denser (Figure 6c) compared to that manufactured by the non-optimized FDM method (Figure 6d). This result was in good agreement with the data on the physical and mechanical properties of the composites (Table 8).

**Figure 7:**
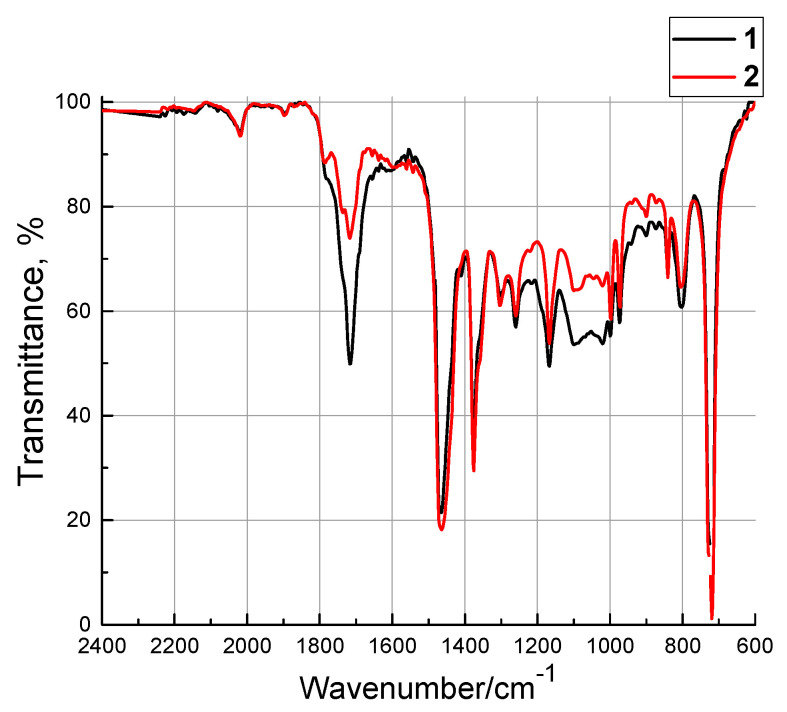
A fragment of the IR spectrum of the composites manufactured by FDM at different part bed temperatures (the red line denotes 100 °C and the black one denotes 120 °C).

The results of IR spectroscopy of the composite manufactured by the non-optimized FDM method at different levels of one factor (the part bed temperature *T*_3Dbed_ of 100 and 120 °C) are presented in Figure 7. The intensity of the characteristic peak located in the range from 1800 to 1650 cm^–1^ enhanced upon 3D printing, which indicated an increase in the number of carbonyl groups.

It should be noted that the carbonyl groups in the composite were as a part of HDPE-g-SMA, which corresponded to the bands of 1725–1705 cm^−1^ (stretching vibrations of the C=O bond) and 1740–1720 cm^−1^ that were relevant to the stretching vibrations of the aldehyde groups. This confirmed the fact that the polymers had been oxidized with atmospheric oxygen, which proceeded both at the end groups and at the CH_2_ ones and was accompanied by the formation of ketones. In addition, partial breaks in the polymer chains of UHMWPE had been possible. This was confirmed by the changes in the IR spectrum in the range 1200–1000 cm^−1^, which corresponded to C–O–C stretching vibrations.

Thus, the polar regions had been formed in the composites manufactured by the non-optimized FDM method that caused the heterogeneous structuring of the polymer chains when part bed table temperature *T*_3Dbed_ (where the solidification process had been taken place) had been risen up to 120 °C. This had deteriorated the physical and mechanical characteristics. This conclusion was consistent with the above results obtained by the Taguchi method. For this reason, higher physical and mechanical characteristics were at the part bed temperature of 100 °C.

### 4.2. Mechanical Test Results

**Figure 8:**
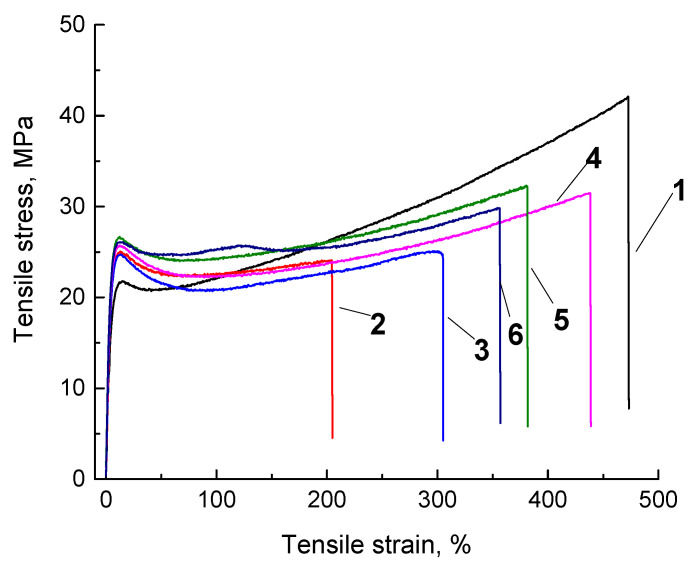
Engineering stress–strain curves for neat UHMWPE (1) and the ‘UHMWPE+17 wt.% HDPE-g-SMA +12 wt.% PP’ composite: fabricated by the non-optimized powder hot pressing (2), fabricated by the non-optimized granules hot pressing (3), fabricated by the optimized granule hot pressing (4), manufactured by the non-optimized FDM method (5), manufactured by the optimized FDM method (6).

Stress–strain curves and summary data on the physical and mechanical properties of the studied composites obtained by various methods are shown in Figure 8 and in Table 8. It follows from the presented data that all composites had comparable yield strength values (from 24.6 up to 26.9 MPa), but their Young’s modulus and elongation at break values were significantly different. The least elongation at break (approximately 200%) had the composite fabricated by powder compression sintering (Figure 8; curve 2). The composite fabricated by granule compression sintering had higher values (approximately 300%) (Figure 8; curve 3). The optimization of the compounding parameters by the Taguchi method enabled improving this parameter up to 480% (Figure 8; curve 4) with the simultaneous increase in Young’s modulus, yield strength, and tensile strength. This unequivocally testified to the correctness of the results of the studies on the optimization of the twin-screw compounding parameters.

As a result of the mechanical tests of the samples manufactured by the non-optimized FDM method, it was revealed that both samples without and after optimization (Figure 8; curves 5 and 6) had smaller values of this parameter in comparison with the sample fabricated by the ‘optimized’ granule compression sintering in terms of elongation at break. A comparison of the curves 5 and 6 showed that the optimized FDM process simultaneously increased density by 0.01 g/cm^3^, Shore D hardness by 1.2, and Young’s modulus by almost 200 MPa. A decrease in yield strength by 1.1 MPa and elongation at break by 25% were established. The authors expectedly associated this fact with the increase in Young’s modulus due to the polymer oxidation upon 3D printing. We also suggested that the optimization by the Taguchi method yielded positive results, since the numerous parameters of the FDM process exerted the opposite effect on the physical and mechanical properties. The carried-out optimization procedure made it possible to identify the factors that ensure the achievement of the maximum physical and mechanical properties.

### 4.3. Tribological Test Results

**Figure 9:**
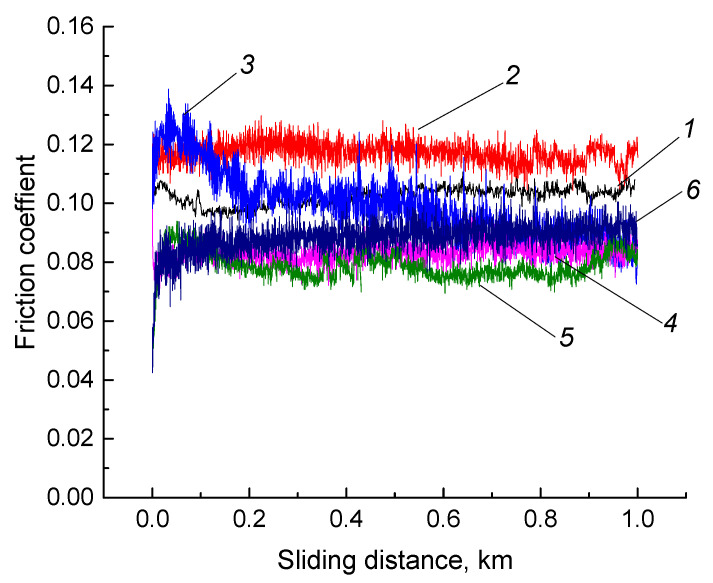
The friction coefficients of neat UHMWPE (1) and the ‘UHMWPE+17 wt.% HDPE-g-SMA +12 wt.% PP’ composite: fabricated by the non-optimized powder hot pressing (2), fabricated by the non-optimized granule hot pressing (3), fabricated by the optimized granules hot pressing (4), manufactured by the non-optimized FDM method (5), manufactured by the optimized FDM method (6).

Since the optimization of the feedstock fabrication and the FDM process parameters was performed without considering the tribological test data, then a comparative study was carried out using the both ‘pin-on-disk’ and ‘block-on-ring’ tribological test schemes. Table 9 presents the data on the wear rate and the friction coefficient ƒ of neat UHMWPE and composites obtained by all three studied methods. The dynamics of the friction coefficient values during tribological tests are shown in Figure 9. In this case, the ‘pin-on-disk’ test scheme had been used when the specific pressure on the friction surface had been high enough (up to 38.2 MPa, which had been much higher than yield strength), and the counterpart tribological contact surface had been the same, when the ‘block-on-ring’ scheme had been applied (described below).

It follows from the data presented in Table 9 that the wear rate and the friction coefficients of the composites fabricated by the conventional granule hot pressing (No. 3 and 4) were significantly lower than that after the powder compression sintering (No. 2; wear rate was 1.2 times, the friction coefficient was one and a half times higher). Wear rates of the composites manufactured by the conventional FDM method (No. 5 and 6) were higher than that after the granule compression sintering 1.1–1.2 times (discussed below when analyzing the topography of the wear track surfaces). However, their friction coefficients were almost the same, i.e., 0.08. Moreover, the sample manufactured by the optimal FDM method showed the most stable friction coefficient values during the entire test period. It should be noted that the composite fabricated by the non-optimized granule hot pressing of Figure 9, curve 3 was characterized by a noticeable oscillation of the friction coefficient magnitude, which gradually decreased with the test time. Apparently, this was due to the gradual running-in of the friction surfaces.

**Figure 10:**
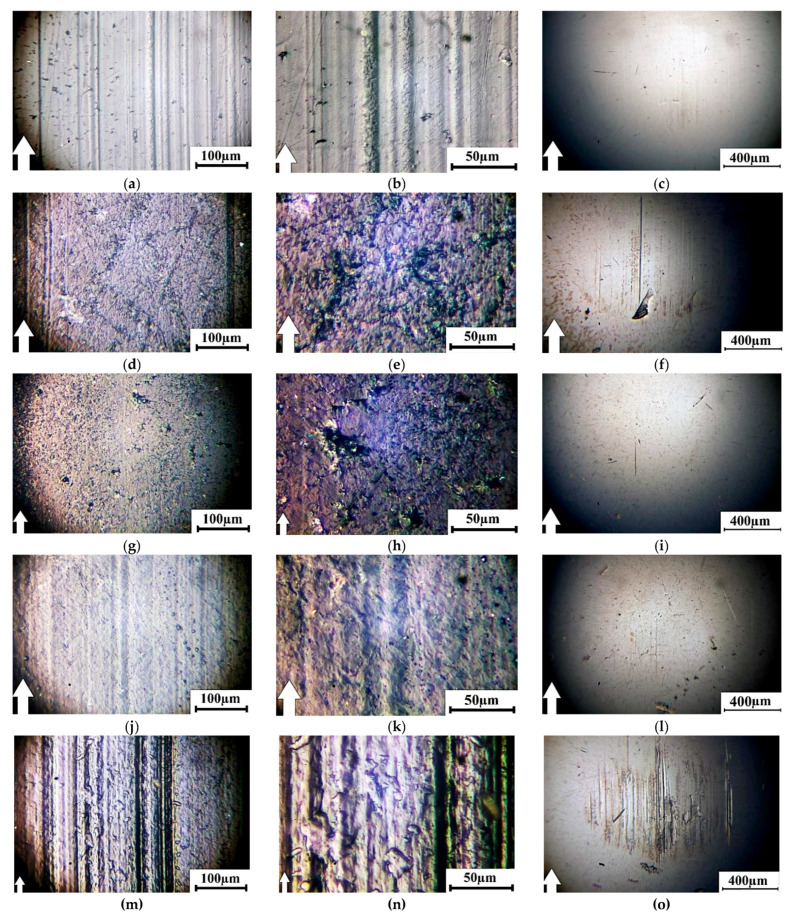

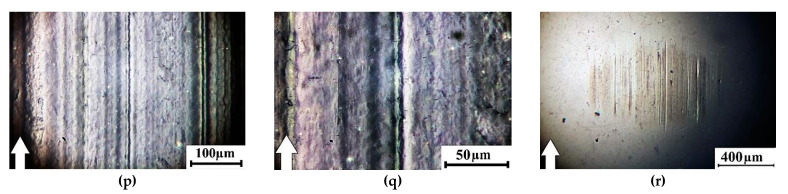
Optical images of the wear track surfaces at 5 N × 0.3 m/s: neat UHMWPE (**a**–**c**) and the ‘UHMWPE + 17 wt.% HDPE-g-SMA + 12 wt.% PP’ composites: fabricated by the optimized powder hot pressing (**d**–**f**); fabricated by the non-optimized granule hot pressing (**g**–**i**); fabricated by the optimized granule hot pressing (**j**–**l**); manufactured by the non-optimized FDM method (**m**–**o**); manufactured by the optimized FDM method (**p**–**r**).

An analysis was made of optical images that characterized the topography of the composite wear surfaces (Figure 10) to identify the reasons why their wear intensity varied. The features of the surface topographies formed due to the friction and wear processes correlated well with the tribological characteristics of the composites (Figure 10 and Table 9). Narrow grooves or ridges of the deformed material oriented in the sliding direction of the steel counterpart were on the wear track of neat UHMWPE (Figure 10a,b). The surface of the steel counterpart was, as expected, smooth and intact (Figure 10c).

The wear track surface of the sample fabricated by the non-optimized powder hot pressing was less uniform due to the nonhomogeneous distribution of the polymer components (Figure 10d,e). Traces of debris in the form of numerous small brown spots were on the surface of the steel counterpart. In turn, there were almost no micro-scratches on the surface of the steel counterpart (Figure 10f).

The wear track surfaces of the samples fabricated by the non-optimized granule hot pressing (both the non-optimized and the optimized) were the smoothest (Figure 10g,h,j,k). The last one’s topology correlated with the homogeneous nature of the formed supermolecular structure. The surface of the steel counterpart was smooth and did not contain traces of sticking debris. This was in good agreement with the minimal wear of such composites. A noticeable difference between the samples fabricated by the conventional and optimized hot pressing of the granules was not found on the images of their wear tracks.

Numerous micro-grooves were on the wear track surfaces of the samples manufactured by the FDM method, particularly by the non-optimized one (Figure 10m). With a high magnification, it was evident that embedded wear particles in the form of ‘inclusions’ of irregular shape were on this surface (Figure 10n). A significant number of micro-scratches and grooves were on the surface of the steel counterpart. The reason for their formation was, most likely, the micro-abrasive effect of debris (Figure 10o). The number of micro-grooves was significantly less on the wear track surfaces of both the sample manufactured by the optimized FDM method (Figure 10p,q) and the steel counterpart (Figure 10r). However, this was not accompanied by a decrease in its wear rate according to Table 9. Nevertheless, the revealed fact further supported the statement that the optimization of the processing parameters by the Taguchi method improved the structure of the sample manufactured by the FDM method.

**Figure 11:**
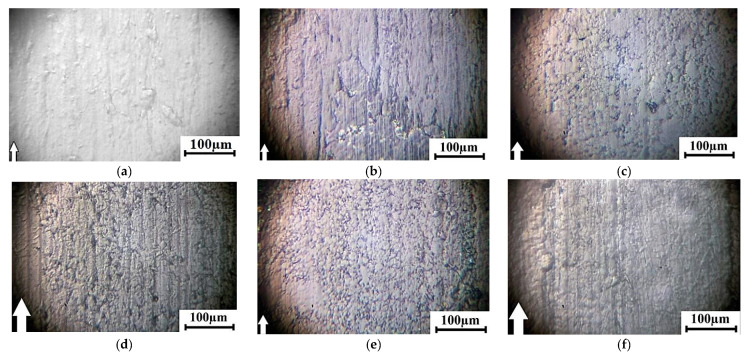
Optical images of the wear track surfaces at 60 N × 0.3 m/s: neat UHMWPE (**a**) and the ‘UHMWPE + 17 wt.% HDPE-g-SMA + 12 wt.% PP’ composites: fabricated by the optimized powder hot pressing (**b**); fabricated by the non-optimized granule hot pressing (**c**); fabricated by the optimized granule hot pressing (**d**); manufactured by the non-optimized FDM method (**e**); manufactured by the optimized FDM method (**f**).

**Figure 12:**
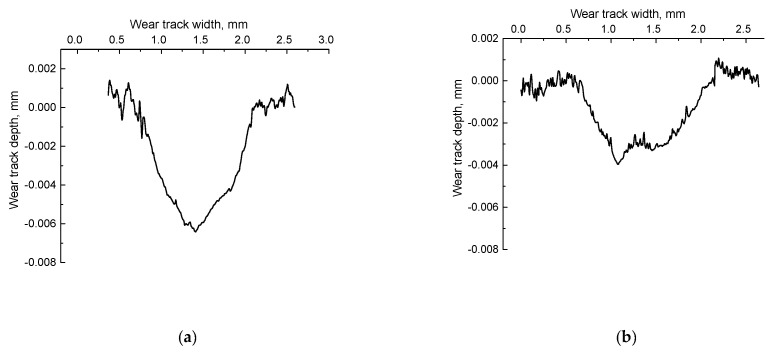

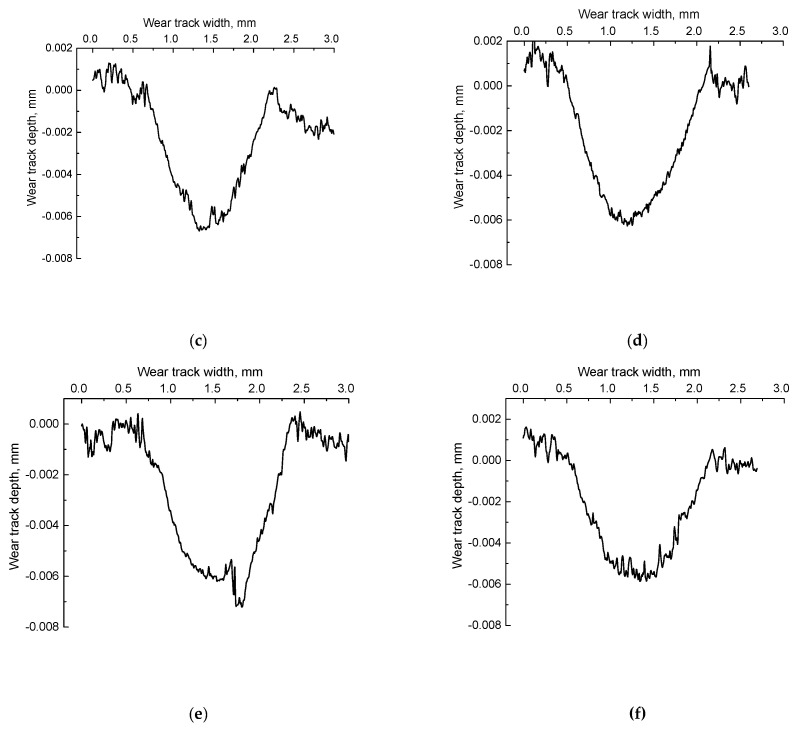
The cross-section wear track profiles at 60 N × 0.3 m/s: neat UHMWPE (**a**) and the ‘UHMWPE + 17 wt.% HDPE-g-SMA + 12 wt.% PP’ composites: fabricated by the optimized powder hot pressing (**b**); fabricated by the non-optimized granule hot pressing (**c**); fabricated by the optimized granule hot pressing (**d**); manufactured by the non-optimized FDM method (**e**); manufactured by the optimized FDM method (**f**).

Then, tests were carried out according to the ‘block-on-ring’ scheme at 60 N and 0.3 m/s to compare the behavior of the composites under other conditions of tribological loading (first of all, with a more distributed counterpart loading and, as a result, lower specific pressure). The results are presented in Table 10, while characteristic photographs of wear track surfaces and wear track profiles are shown in Figure 11 and Figure 12, respectively.

All studied composites were comparable with neat UHMWPE fabricated by hot pressing in terms of ‘net’ wear considering the elastic recovery upon 24 h after the tribological tests (Table 10). At the same time, the sample fabricated by the powder compression sintering (0.37 × 10^−6^ mm^3^/N × m) was the least worn out. It was 1.3 times lower than that of neat UHMWPE with the worst mechanical properties. The wear of the sample manufactured by the optimized method was the highest (0.63 × 10^−6^ mm^3^/N × m). It was just 1.3 times higher than that of neat UHMWPE. The obtained data showed that the optimization of the compounding and the FDM process parameters almost did not have a noticeable effect on the wear rate of the samples. However, the values of the elastic recovery of the wear track were almost the same for both samples obtained by the optimized granule hot pressing and by the optimized FDM method. This testified in favor of the structure uniformity of the composites manufactured by the optimized FDM method.

The photographs of the wear track surfaces shown in Figure 11 markedly differed from those for the ‘pin-on-disk’ test scheme (Figure 10). The surfaces of all studied samples were quite smooth, even the composite fabricated by the powder compression sintering, which had maximum both structural heterogeneity and wear resistance (Figure 11b and Figure 12b). These results were consistent with the contact profilometry of the wear tracks (Figure 12). The topography of the wear track surfaces on the samples obtained by the granule compression sintering and by the FDM methods reflected the uniform dispersion of PP in the UHMWPE matrix. Therefore, the surfaces were ‘spotted’ (Figure 11c–f). The distributed counterpart loading, as well as the low degree of wear that did not cause the formation of a noticeable amount of debris, resulted in the presence of micro-scratches and grooves on the sample surfaces.

## 5. Discussion

Few data on the properties of UHMWPE samples manufactured by 3D printing were published [34]. UHMWPE possessed an ultra-high viscosity (low melt flow rate), which drastically reduced the efficiency of the conventional continuous plastics processing (in the molten state), including additive manufacturing (namely FDM). Attempts were mainly made to overcome this problem and adapt the material for the FDM or the SLS processes by mixing UHMWPE with high-density polyethylene (HDPE) at its high concentration (up to 60%). As a result, such composites possessed the properties of HDPE rather than those of UHMWPE.

The mechanical and tribological properties (tensile strength, yield strength, elongation at break, the Young’s modulus, the friction coefficient, and wear resistance) of the extruded UHMWPE-based composites designed by the authors and manufactured by the FDM method were at least not inferior to similar characteristics of the ones fabricated by compression sintering. This was due to the formation of the more uniform supermolecular structure due to the feedstock processing in the twin-screw extruder. These results were partially presented in a monograph chapter [35].

According to the results of the studies of the structure and the operational properties of the three-component UHMWPE-based composites obtained by the twin-screw compounding and the FDM method using the parameters optimized by the Taguchi method based on their mechanical characteristics, the authors stated the following. The determined optimal parameters (factors) of the liquid-phase blending of the polymer components enabled simultaneously increasing their mechanical and tribological characteristics. The preset optimal FDM method parameters had improved the basic physical and mechanical properties of the composites (density, hardness, Young’s modulus). However, their tribological properties decreased slightly. According to the authors, this was due to the stronger oxidation.

The manufacture of products from polymer composites by various methods made it possible to achieve the high physical, mechanical, and tribological properties. The effects of the studied factors and their levels on the mentioned characteristics were compared in this paper. For example, the use of the two-screw compounding of the composite components resulted in the noticeable improvements in all its properties. However, most of these characteristics decreased during the FDM process. The aim of this paper was to find appropriate levels of the process factors that ensured the formation of the composites possessing the enhanced physical and mechanical properties, as well as to understand the reasons for these effects.

Shear strains were upon processing in the twin-screw extruder. They intensified the interaction of the composite components and enabled forming the more uniform supermolecular structure due to the mutual penetration of polymer chains. The factors such as the extrusion temperature, the extruder screw rotation speed, and the amount of the extruded material played a decisive role for the efficient mixing and the polymer entropy increasing. This was reflected in the achievement of the high physical and mechanical properties.

The designed composite was intended for the manufacture of products with high tribological characteristics. In addition, they had to possess improvements in both the Young’s modulus and yield strength. The 3D printing process was more complicated than compression sintering. Nevertheless, it was necessary to determine the appropriate process parameters to develop the procedure. In particular, the formation of microcracks should be noted to assess the effect of 3D printing on the supermolecular structure ordering. The incorrectly determined part bed temperature could contribute to this fact. In addition, the authors observed various thicknesses and the filament integrity violation during the FDM process, which was due to the insufficient fluidity of the molten polymer. The increase in the part bed temperature caused the development of two mutually affecting processes: the oxidation intensification and the polymer destruction as a result. At the same time, the sintering ability of the polymer composite increased due to its better spreading. Thus, the increase in the part bed temperature contributed to the healing of defects due to the low extrudability of the polymer feedstock. According to the authors, fusible links of polymer chains could move under the influence of a higher temperature, causing the effective redistribution (or relaxation) of mechanical stresses. As a result, the Young’s modulus and tensile strength could be increased.

In general, according to the data obtained for the twin-screw compounding, it was necessary to ensure the following: (1) a sufficient level of temperature for the homogeneous combination of the polymer mixture components, and (2) the extruder screw rotation speed, enabling their better combining (dispersing). Moreover, the increase in the number of passes (mixing) also stimulated the composite homogeneity improvement. A key factor influencing the 3D printing process was sintering, which, however, proceeded under extremely nonequilibrium conditions. It was shown in the paper that expanding the levels of other FDM process factors enabled providing their more significant effect. The studied composite did not allow significantly expanding the range of their values. However, the authors (so far) were able to effectively implement the 3D printing of UHMWPE only with its use.

It should be noted once again as the conclusion that each factor can be significant for both compounding products and their manufacturing. However, the best physical and mechanical properties are achieved only by the formation of the optimal (primarily homogeneous) composite superstructure.

By the way of summarizing, the following remarks are noteworthy. The mechanical and tribological properties (tensile strength, yield strength, elongation at break, the Young’s modulus, the friction coefficient, and wear resistance) of the extruded UHMWPE-based composites manufactured by the FDM method have exceeded the similar characteristics of the ones fabricated by compression sintering. The reason has been the formation of the more uniform supermolecular structure of the polymer composite. Such composites can be applied for the manufacture of complex-shaped friction units operating at subzero temperatures, in aggressive environments, as well as in a wide range of loads and sliding speeds. Finally, the designed composites are prospects for conventional plastics processing methods (for example, single-screw extrusion in the production of pipes and other large-scale products) due to their satisfactory extrudability.

## 6. Conclusions

1. The values of the factors (processing parameters) were determined using the Taguchi method. They enabled to achieve the maximum values of the mechanical characteristics of the ‘UHMWPE + 17 wt.% HDPE-g-SMA + 12 wt.% PP’ composites manufactured by the FDM method. The ranking of the FDM process parameters by their effect degree was carried out:
  - As the optimum extrusion temperature T_extr2scr_, its maximum investigated value of 220 °C had to be taken. In this case, the strength characteristics, such as Young’s modulus and tensile strength, had the highest values. The optimal extruder screw rotation speed *v*_extr2scr_ had to be between 50 and 60 rpm. The physical and mechanical properties of the composites deteriorated at lower values of this factor. From the standpoint of estimating the number of revolutions, the choice of the most suited value was the result of a compromise between the time spent on the mixing and the composite mechanical characteristics. The single processing of the powder mixture in the twin-screw extruder did not enable them to mix it well enough.
  - A part bed temperature *T*_3Dbed_ of 100 °C, printing speed *v*_3D_ of 20 mm/s, and micro-extruder temperature *T*_3Dextr_ of 170 °C were the optimal parameters of the FDM process to achieve the highest values of the physical and mechanical properties of the composites. The optimal micro-extruder screw rotation speed *f*_3D_, which was proportional to the amount of the supplied material, was the factor that had the minimal effect. It had to be taken equal to the average of the used values (12.12 rpm).
2. The samples fabricated by the optimized both compression sintering and the FDM process had a uniform finely dispersed structure with a high degree of dispersion of polypropylene as a plasticizing component. The supermolecular structure of the samples manufactured by the FDM process was, as expected, ‘looser’ than that fabricated by compression sintering. This was consistent with their density and corresponded to the processing features. However, the quantitative difference in the density values was rather small (0.94 g/cm^3^ after compression sintering and 0.93 g/cm^3^ after the FDM process).
3. It was shown that the formation of the finely dispersed homogeneous structure of the three-component polymer composite, subjected to the twin-screw compounding to fabricate the feedstock and the single-screw extrusion during the FDM process, provided high mechanical and tribological (with dry sliding friction on the steel counterpart) properties. The maximum Young’s modulus (1145 MPa compared to 965 MPa) had the composite manufactured by the FDM method, while the samples fabricated by granule compression sintering had the maximum wear resistance during dry sliding friction (0.53 × 10^−6^ mm^3^/N×m compared to 0.63 × 10^−6^ mm^3^/N × m).
4. Since the processing parameters (factors) were optimized over the mechanical test results, the wear resistance of the FDM composites was slightly lower than that fabricated by granule compression sintering. This was due to the less dense structure of the composite manufactured by the FDM method and, consequently, the lighter wear debris separation. The latter exerted a microabrasive effect on the steel counterpart. This resulted in the formation of micro-scratches and grooves on its surface. Nevertheless, the optimization of the compounding and 3D-printing parameters was carried out using the Taguchi method. The achieved physical and mechanical and tribological properties make it possible to recommend both the composites and the process parameters for the implementation to produce parts of friction units by additive manufacturing.

## Figures and Tables

**Table 1:** Temperatures of the five zones in the twin-screw extruder, °C.

1st Zone	2nd Zone	3rd Zone	4th Zone	Die	Designation in the Text
90	210	245	245	230	230
85	200	235	235	220	220
83	195	225	225	210	210

**Table 2:** Matching of the factors and their levels for the twin-screw compounding in accordance with the Taguchi L9 orthogonal matrix for three-factor experiments.

	Level/Factor	The Amount of the Extruded Material (*N*_extr_)	Extrusion Temperature (*T*_extr2scr_), °C	The Extruder Screw Rotation Speed (υ_extr2scr_), rpm
Experiment Number				
1		1/1	1/210	1/40
2		1/1	2/220	2/50
3		1/1	3/230	3/60
4		2/2	1/210	2/50
5		2/2	2/220	3/60
6		2/2	3/230	1/40
7		3/3	1/210	3/60
8		3/3	2/220	1/40
9		3/3	3/230	2/50

**Table 3:** The physical and mechanical properties of the ultra-high molecular weight polyethylene (UHMWPE)-based composites fabricated by hot pressing of the granulate with varying the twin-screw extrusion parameters.

Composition Number	Density (*ρ*), g/cm^3^	Shore (*D*) Hardness	Young’s Modulus (*G*), MPa	Yield Strength (σ_Y_), MPa	Tensile Strength (σ_T_), MPa	Elongation at Break (ε), %
Neat UHMWPE	0.934	57.7 ± 0.6	711 ± 35	21.6 ± 0.6	42.9 ± 1.5	485 ± 28
1	0.954	58.9 ± 0.4	901 ± 37	25.4 ± 0.9	30.1 ± 0.9	427 ± 49
2	0.948	58.2 ± 0.4	959 ± 42	25.1 ± 0.5	33.8 ± 1.4	469 ± 57
3	0.949	57.0 ± 0.6	987 ± 48	25.7 ± 0.6	24.9 ± 1.2	279 ± 52
4	0.945	57.6 ± 0.5	947 ± 40	25.9 ± 0.5	30.2 ± 1.2	372 ± 40
5	0.950	57.7 ± 0.2	989 ± 38	26.1 ± 0.6	33.5 ± 1.9	444 ± 38
6	0.944	57.6 ± 0.3	943 ± 25	25.7 ± 0.5	23.7 ± 0.8	241 ± 32
7	0.943	58.2 ± 0.5	1000 ± 22	25.9 ± 0.9	34.1 ± 1.8	440 ± 56
8	0.946	57.8 ± 0.4	980 ± 43	25.8 ± 0.9	36.9 ± 1.5	497 ± 53
9	0.943	57.7 ± 0.7	984 ± 62	25.2 ± 0.8	31.5 ± 1.2	423 ± 43

**Table 4:** The total effect of the factors on the maximum values of the mechanical properties of the composites compounded by the twin-screw extrusion.

Properties	The Extruder Screw Rotation Speed, υ_extr2scr_	Extrusion Temperature, *T*_extr2scr_	The Amount of the Extruded Material, *N*_extr_
Young’s Modulus	1	2	3
Yield Strength	2	3	1
Tensile Strength	3	1	2
Elongation at Break	3	1	2
Total	9	7	8
The Optimal Value	55 rpm	220 °C	Triple Extrusion

**Table 5:** Matching of the factors and their levels in accordance with the Taguchi L9 orthogonal matrix for four-factor experiments to determine the optimal 3D printing parameters.

Run	Control Factor and Levels			
	The Micro-Extruder Screw Rotation Speed (*f*_3D_), rpm	Part Bed Temperature (*T*_3Dbed_), °C	Printing Speed (*v*_3D_), mm/s	Micro-Extruder Temperature (*T*_3Dextr_), °C
1	1/11.76	1/100	1/10	1/170
2	1/11.76	2/110	2/20	2/180
3	1/11.76	3/120	3/30	3/190
4	2/12.12	1/100	2/20	3/190
5	2/12.12	2/110	3/30	1/170
6	2/12.12	3/120	1/10	2/180
7	3/12.66	1/100	3/30	2/180
8	3/12.66	2/110	1/10	3/190
9	3/12.66	3/120	2/20	1/170

**Table 6:** The physical and mechanical properties of the UHMWPE-based composites fabricated by granulate hot pressing with varying the Fused Deposition Modeling (FDM) process parameters.

Composition Number	Density (*ρ*), g/cm^3^	Shore (*D*) Hardness	Young’s Modulus (*G*), MPa	Yield Strength (σ_Y_), MPa	Tensile Strength (σ_T_), MPa	Elongation at Break (ε), %
Neat UHMWPE	0.934	57.7 ± 0.6	711 ± 35	21.6 ± 0.6	42.9 ± 1.5	485 ± 28
1	0.924	59.5 ± 0.7	994 ± 18	25.9 ± 0.8	32.3 ± 1.9	435 ± 71
2	0.927	60.2 ± 2.2	975 ± 15	25.5 ± 0.2	29.3 ± 1.3	424 ± 39
3	0.921	58.8 ± 3.2	956 ± 51	25.4 ± 0.5	24.9 ± 1.8	340 ± 59
4	0.929	59.3 ± 2.3	947 ± 34	25.1 ± 0.4	28.9 ± 1.1	415 ± 41
5	0.923	59.3 ± 4.1	951 ± 61	25.5 ± 0.6	29.7 ± 1.7	422 ± 76
6	0.911	59.3 ± 4.1	957 ± 55	25.2 ± 0.3	21.2 ± 1.2	218 ± 94
7	0.923	59.9 ± 3.9	1002 ± 36	25.6 ± 0.2	30.5 ± 2.2	425 ± 54
8	0.915	60.1 ± 3.8	949 ± 46	25.6 ± 0.6	23.1 ± 1.5	251 ± 46
9	0.928	59.1 ± 3.9	940 ± 35	24.9 ± 0.7	30.1 ± 0.7	422 ± 66

**Table 7:** The total effect of the factors on the maximum values of the mechanical properties for the FDM method.

	Factor	The Micro-Extruder Screw Rotation Speed (*f*_3D_), rpm	Part Bed Temperature (*T*_3Dbed_), °C	Printing Speed (*v*_3D_), mm/s	Micro-Extruder Temperature (*T*_3Dextr_), °C
Mechanical Properties					
Young’s Modulus		3	1	4	2
Yield Strength		3	1	2	4
Tensile Strength		4	1	3	2
Elongation at Break		4	2	1	3
Total		14	5	10	11
The Optimal Value		Not Found	100 °C	20 mm/s	170 °C

**Table 8:** The mechanical properties of neat UHMWPE and the ‘UHMWPE+17 wt.% HDPE-g-SMA +12 wt.% PP’ Composites.

No.	Filler Composition. %	Density (*ρ*). g/cm^3^	Shore (*D*) Hardness	Young’s Modulus (*G*). MPa	Yield Strength (σ_Y_). MPa	Tensile Strength (σ_T_). MPa	Elongation at Break (ε). %
1	Neat UHMWPE	0.93	57.7 ± 0.6	711 ± 35	21.6 ± 0.6	42.9 ± 1.5	485 ± 28
2	UHMWPE+17 wt.% HDPE-g-SMA +12 wt.% PP Fabricated by the Powder Compression Sintering	0.94	60.4 ± 0.7	814 ± 41	24.9 ± 0.3	22.9 ± 1.2	203 ± 28
3	UHMWPE+17 wt.% HDPE-g-SMA +12 wt.% PP Fabricated by the Granule Compression Sintering	0.95	59.2 ± 0.5	923 ± 28	24.6 ± 0.3	23.5 ± 2.6	307 ± 36
4	UHMWPE+17 wt.% HDPE-g-SMA +12 wt.% PP Fabricated by the Optimized Granule hot Pressing	0.94	57.6 ± 0.4	965 ± 24	25.6 ± 0.3	32.8 ± 2.2	438 ± 36
5	UHMWPE+17 wt.% HDPE-g-SMA +12 wt.% PP Manufactured by the Non-Optimized FDM Method	0.92	55.5 ± 0.6	948 ± 58	26.9 ± 0.4	31.1 ± 0.8	381 ± 24
6	UHMWPE+17 wt.% HDPE-g-SMA +12 wt.% PP Manufactured by the Optimized FDM Method	0.93	56.7 ± 0.8	1145 ± 41	25.8±0.4	30.1 ± 1.3	356 ± 42

**Table 9:** The tribological properties of neat UHMWPE and the UHMWPE-based composites (the ‘Pin-on-Disk’ test scheme).

No.	Filler Composition, %	Wear Rate, 10^−5^ mm^3^/N×m	Friction Coefficient, ƒ
1	Neat UHMWPE	2.72 ± 0.48	0.10 ± 0.01
2	UHMWPE+17 wt.% HDPE-g-SMA +12 wt.% PP Fabricated by the Powder Compression Sintering	2.64 ± 0.52	0.12 ± 0.01
3	UHMWPE+17 wt.% HDPE-g-SMA +12 wt.% PP Fabricated by the Granule Compression Sintering	2.22 ± 0.36	0.08 ± 0.01
4	UHMWPE+17 wt.% HDPE-g-SMA +12 wt.% PP Fabricated by the Optimized Granule Hot Pressing	2.18 ± 0.29	0.08 ± 0.01
5	UHMWPE+17 wt.% HDPE-g-SMA +12 wt.% PP Manufactured by the Non-Optimized FDM Method	2.50 ± 0.31	0.08 ± 0.01
6	UHMWPE+17 wt.% HDPE-g-SMA +12 wt.% PPManufactured by the Optimized FDM Method	2.55 ± 0.28	0.09 ± 0.01

**Table 10:** The tribological properties of neat UHMWPE and the UHMWPE-based composites (the ‘‘Block-on-Ring’’ test scheme).

	Filler Composition, %	Wear rate, 10^–6^ mm^3^/N m Excluding Elastic Recovery	Wear rate, 10^–6^ mm^3^/N m After 24 h	Elastic Recovery, %
1	Neat UHMWPE	1.34 ± 0.48	0.49 ± 0.21	63
2	UHMWPE+17 wt.% HDPE-g-SMA +12 wt.% PP Fabricated by the Powder Compression Sintering	0.69 ± 0.12	0.37 ± 0.06	46
3	UHMWPE+17 wt.% HDPE-g-SMA +12 wt.% PP Fabricated by the Granule Compression Sintering	1.00 ± 0.11	0.56 ± 0.03	44
4	UHMWPE+17 wt.% HDPE-g-SMA +12 wt.% PP Fabricated by the Optimized Granule hot Pressing	0.97 ± 0.08	0.53 ± 0.02	45
5	UHMWPE+17 wt.% HDPE-g-SMA +12 wt.% PP Manufactured by the Non-Optimized FDM Method	1.09 ± 0.07	0.60 ± 0.04	45
6	UHMWPE+17 wt.% HDPE-g-SMA +12 wt.% PP Manufactured by the Optimized FDM Method	1.10 ± 0.11	0.63 ± 0.03	43
